# Serial fecal calprotectin as a screening tool for subclinical ileitis in patients with idiopathic uveitis or scleritis: a prospective cohort study

**DOI:** 10.3389/fmed.2026.1968285

**Published:** 2026-10-26

**Authors:** Cristina Lavilla Olleros, Luis Menchén, Joana Ramos-Ferreira, Beatriz Merino Rodríguez, José María de Miguel-Yanes, Rosa Gutierrez Bonet, Javier Torresano Rodríguez, Cristina Ausín García, Carolina Puertas López, Luis Alvarez-Sala Walther, Lucía Ibares-Frías

**Affiliations:** 1 Department of Medicine, Faculty of Medicine, Complutense University of Madrid (UCM), Madrid, Spain; 2 Department of Internal Medicine, Gregorio Marañón University General Hospital (HGUGM), Madrid, Spain; 3 Centre for Immune-Mediated Diseases (CEIMI), Gregorio Marañón University General Hospital (HGUGM), Madrid, Spain; 4 Instituto de Investigacion Sanitaria Gregorio Marañón (IiSGM), Madrid, Spain; 5 Department of Gastroenterology, Gregorio Marañón University General Hospital (HGUGM), Madrid, Spain; 6 Department of Geriatric, Gregorio Marañón University General Hospital (HGUGM), Madrid, Spain; 7 Department of Clinical Biochemistry, Gregorio Marañón University General Hospital (HGUGM), Madrid, Spain; 8 Department of Ophthalmology, Gregorio Marañón University General Hospital (HGUGM), Madrid, Spain

**Keywords:** biomarker, fecal calprotectin, gut-eye axis, multidisciplinary management, scleritis, subclinical ileitis, subclinical inflammatory bowel disease, uveitis

## Abstract

Idiopathic uveitis accounts for 27%–51% of uveitis cases in Europe and the United States. Subclinical inflammatory bowel disease (IBD) may remain undiagnosed in patients with idiopathic uveitis or scleritis despite shared immunopathogenic mechanisms and the absence of digestive symptoms. Early identification may enable intervention within the inflammatory window of opportunity, yet subclinical ileitis has not been systematically explored in this population. We evaluated the diagnostic performance of serial fecal calprotectin for identifying subclinical inflammatory bowel disease in a prospective cohort of 69 patients with idiopathic uveitis or scleritis, using a baseline measurement repeated at 6–12 months if negative or at three months if positive, defining four longitudinal patterns (A–D). Subclinical ileitis was confirmed by capsule endoscopy and/or colonoscopy in 5 of the 62 patients with an evaluable outcome (8.1%). Baseline fecal calprotectin was significantly higher in patients with confirmed ileitis (*p* = 0.024), a difference that became more pronounced at the second determination (*p* = 0.002). The pattern of persistent elevation (subgroup D) showed the best diagnostic balance: sensitivity 100% (exact 95% CI: 47.8–100.0), specificity 82.5% (95% CI: 70.1–91.3), negative predictive value 100% (95% CI: 92.5–100.0), and positive likelihood ratio 5.7 (95% CI: 3.25–10.01). ROC curve analysis showed a numerically higher discriminative capacity for the second calprotectin determination (AUC: 0.932; 95% CI: 0.850–1.000) than for the baseline measurement (AUC: 0.809; 95% CI: 0.652–0.966), although a formal DeLong comparison between both c-statistics found no statistically significant difference (*p* = 0.242). This exploratory, hypothesis-generating cohort suggests that a persistently elevated fecal calprotectin trajectory identifies a subgroup of patients with idiopathic uveitis or scleritis who may warrant digestive evaluation; because subgroups A and C were not endoscopically verified, the reported sensitivity and negative predictive value are estimates conditioned on this verification scheme and on only five events, not validated evidence that a negative trajectory excludes subclinical ileitis. Confirmation in larger, prospectively verified, multicenter cohorts is required before any screening recommendation can be made.

## Introduction

Idiopathic uveitis, defined by the absence of an identifiable systemic etiology, accounts for 27%–51% of uveitis cases in Europe and the United States, representing the largest single etiological category (1). Uveitis and scleritis can be understood as clinical phenotypes in which loss of ocular tolerance, activation of innate and adaptive immunity, genetic susceptibility, and environmental influences converge, supporting the interpretation of ocular inflammation not merely as a localized event but as a possible manifestation of systemic immune-mediated disease. Among the systemic contexts of greatest interest is inflammatory bowel disease (IBD), given its capacity to combine digestive inflammation, extraintestinal manifestations, and biomarkers of inflammatory activity.

IBD, comprising Crohn's disease, ulcerative colitis and indeterminate colitis, is a chronic, polygenic, and multifactorial condition resulting from the interaction of genetic factors, alterations in the gut microbiome, mucosal barrier dysfunction, and an inappropriate immune response to luminal antigens (2). The prevalence exceeds 7 million people and, in Spain, close to 1% of the population (2, 3). Its therapeutic paradigm has evolved toward treat-to-target strategies aimed at endoscopic and histological remission, with fecal calprotectin as a key non-invasive monitoring tool, in a shift from a step-up approach toward an early, individualized top-down strategy. In this context, the concept of the inflammatory window of opportunity has gained relevance: the early period during which intensive therapeutic intervention can significantly modify the natural history of the disease; the European Crohn's and Colitis Organization (ECCO) and the specific recommendations of the Spanish Working Group on Crohn's Disease and Ulcerative Colitis (GETECCU) guidelines emphasize the importance of early control of inflammation to avoid the point of no return beyond which structural damage becomes irreversible (3–6).

IBD frequently extends beyond the gut, with at least one joint, ocular, or skin extraintestinal manifestation (EIM) reported in approximately 24% of patients, with a higher prevalence in Crohn's disease (35%) than in ulcerative colitis (27%) (7). Ocular involvement (episcleritis, scleritis, and uveitis) affects 4%–12% of patients and ranks as the third most frequent EIM after musculoskeletal and mucocutaneous disease; episcleritis typically tracks intestinal activity, whereas uveitis and scleritis more often follow an independent course (7–12).

Ocular inflammation may precede the diagnosis of intestinal disease. In a cohort of 38,805 patients with ocular inflammation, King et al. found a significantly increased subsequent risk of IBD (HR: 2.25), with a mean interval of more than two years between ocular and intestinal diagnoses (13). A bidirectional Mendelian randomization study by Zhong et al. further supported a predominantly unidirectional relationship between IBD and uveitis, with genetic susceptibility to IBD associated with an increased risk of uveitis (OR: 1.14) (14, 15).

Despite this evidence linking ocular inflammation to underlying intestinal disease, in the specific subgroup of patients with idiopathic uveitis or scleritis (i.e., without an established systemic diagnosis), the possibility of subclinical ileitis has not been systematically explored in the literature.

Calprotectin, a heterodimer formed by the S100A8 and S100A9 proteins released by activated neutrophils and monocytes, is involved in inflammatory signaling and has been implicated in both systemic and local inflammatory responses (16–18). In the digestive setting, fecal calprotectin is a well-established non-invasive biomarker for detecting and monitoring intestinal inflammation in IBD (2–4). These characteristics support its evaluation as a screening tool for subclinical intestinal inflammation in patients with idiopathic ocular inflammation. Its utility has also been investigated in other immune-mediated conditions with intestinal involvement, including spondyloarthritis and Behçet's disease (19–21). We therefore evaluated the diagnostic performance of serial fecal calprotectin for identifying subclinical ileitis in patients with idiopathic uveitis or scleritis without digestive symptoms.

## Methods

### Study design and population

An observational cohort study was conducted, with prospective sample collection and longitudinal follow-up, in adult patients with idiopathic uveitis and/or scleritis attended at the Center for Immune-Mediated Diseases (CEIMI) at the Gregorio Marañón University General Hospital (HGUGM, Madrid, Spain) between March 2023 and March 2026. The patient was the unit of analysis. The study is reported in accordance with the STROBE statement for observational studies.

Adults older than 18 years were consecutively included if they met all of the following criteria: a diagnosis of idiopathic non-infectious ocular inflammation, defined as ocular inflammation for which no associated systemic disease or infectious etiology had been identified after standard diagnostic evaluation, regardless of ocular inflammatory activity at the time of inclusion; no prior diagnosis of inflammatory bowel disease (IBD); no digestive symptoms suggestive of IBD or gastrointestinal infection; and signed informed consent.

This etiological work-up included detailed ophthalmological assessment with anatomical classification and activity grading according to SUN criteria, review of systemic symptoms and prior diagnoses, and targeted laboratory and/or imaging investigations to exclude infectious causes and associated systemic immune-mediated diseases, in line with established diagnostic approaches for adult uveitis (1, 22).

All patients were followed up in the complex uveitis clinic, which is jointly managed by the Ophthalmology and Internal Medicine departments at CEIMI. All patients had recurrent, chronic, or persistent uveitis according to the Standardized Uveitis Nomenclature (SUN) (23, 24), as shown in Table 1 and, for the five confirmed cases of ileitis, in Table 6.

**Table 1:** Baseline characteristics of the study population: demographic and clinical data, treatment, laboratory parameters, ocular characteristics, and fecal calprotectin (*N* = 69).

Variable	Value	Valid *n*
General demographic and clinical data		
Age, years	46.4 ± 15.9	69
Sex, male/female	31 (44.9%)/38 (55.1%)	69
Smoking status, current/former/never	16 (23.2%)/2 (2.9%)/51 (73.9%)	69
Treatment		
NSAID use, yes	9 (13.0%)	69
PPI use, yes	29 (42.0%)	69
Systemic corticosteroid therapy (oral prednisone and/or methylprednisolone pulses), yes	26 (37.7%)	69
Conventional or biologic DMARD therapy^a^, yes	30 (43.5%)	69
Active immunosuppression, yes	36 (52.2%)	69
Laboratory parameters		
HLA-B27^b^, positive/negative	4 (7.5%)/49 (92.5%)	53
Ocular characteristics		
Localization of ocular inflammation	Anterior 25 (36.2%)	69
	Intermediate 11 (15.9%)	
	Posterior 10 (14.5%)	
	Panuveitis 9 (13.0%)	
	Scleritis 14 (20.3%)	
Unilateral/bilateral involvement	Unilateral 31 (44.9%); Bilateral 38 (55.1%)	69
Active ocular disease at inclusion, yes/no^e^	Yes 53 (76.8%)	69
	No 16 (23.2%)	
IOP, mmHg	14.43 ± 3.16	69
Macular thickness, affected eye, µm	264.00 (250.75–282.50)	66
RNFL^c^ thickness, affected eye, µm	108.00 (105.00–119.00)	65
Duration of ocular disease, months	40 (8–88.5)	67
Episode type at inclusion, recurrence/first episode	Recurrence 69 (100.0%)/First episode 0 (0.0%)	69
SUN uveitis course classification, recurrent/chronic/persistent	Recurrent 39 (56.5%); Chronic 11 (15.9%); Persistent 19 (27.5%)	69
BCVA^d^ LogMAR, worse eye	0.10 (0.00–0.30)	69
Fecal calprotectin		
Baseline fecal calprotectin, µg/g	36.00 (8.66–74.80)	69
Fecal calprotectin, 2nd determination, µg/g	33.10 (12.25–93.90)	62

NSAID, non-steroidal anti-inflammatory drug; PPI, proton pump inhibitor; DMARD, disease-modifying antirheumatic drug; HLA-B27, human leukocyte antigen B27; IOP, intraocular pressure; RNFL, retinal nerve fiber layer; BCVA, best-corrected visual acuity. Quantitative variables are expressed as mean ± standard deviation or median (interquartile range), depending on their distribution (Shapiro–Wilk or Kolmogorov–Smirnov test, as appropriate); qualitative variables are expressed as absolute frequency and percentage.

a Includes patients receiving synthetic DMARDs (25/69, 36.2%) and/or biologic DMARDs (13/69, 18.8%); categories are not mutually exclusive (8 patients received both). Expressed as overall exposure (yes/no); the analytic database does not include columns for individual drugs (methotrexate, azathioprine, cyclosporine, mycophenolate mofetil, adalimumab, infliximab, tocilizumab), so this breakdown is not presented.

b HLA-B27 testing was not performed in all patients (53/69).

c Retinal nerve fiber layer thickness, obtained using the “3D Disc-RNFL Total Thickness” scan protocol (Topcon 3D OCT-1 Maestro).

d Visual acuity converted to its LogMAR equivalent from the original decimal recording.

e Active ocular disease at inclusion was dichotomized from the clinical status recorded at inclusion: patients classified as active with treatment or active without treatment were considered active, whereas those classified as inactive with treatment or inactive without treatment were considered inactive.

Charlson comorbidity index, presence of another immune-mediated disease, and anterior/posterior segment complications are presented in Supplementary Table 1.

Patients who, despite meeting the above criteria, presented any of the following conditions were excluded: a fecal calprotectin determination performed less than 5 years before inclusion; digestive symptoms suggestive of IBD or gastrointestinal infectious disease prior to fecal sample collection; a prior diagnosis of IBD; active primary neoplasia of the gastrointestinal tract; participation in a clinical trial at any time during the study; or pregnancy at any point between inclusion and study completion. The first of these criteria was applied because a previous determination may already have prompted gastroenterological evaluation and would therefore have removed prevalent subclinical disease from the cohort.

No formal sample size calculation was performed; all eligible patients attended during the study period were consecutively included.

Ocular inflammation was classified anatomically (anterior, intermediate, or posterior uveitis, panuveitis, or scleritis) and its activity graded according to the Standardization of Uveitis Nomenclature (SUN) criteria (23, 24). Best-corrected visual acuity was recorded in logMAR, and macular and retinal nerve fiber layer thickness were measured by optical coherence tomography. Accordingly, this study should be regarded as exploratory and hypothesis-generating rather than as a definitive validation of a screening algorithm.

### Ethics approval

The study was approved by the Research Ethics Committee for Medicinal Products (CEIm) of the HGUGM (record 06/23, dated March 28, 2023) and was conducted in accordance with the principles of the Declaration of Helsinki, Spanish Law 14/2007 on Biomedical Research, Spanish Law 41/2002 on patient autonomy, Regulation (EU) 2016/679 (GDPR), and Spanish Organic Law 3/2018 on Personal Data Protection and Guarantee of Digital Rights; the collection and storage of biological samples complied with Royal Decree 1716/2011. The study does not constitute a clinical trial with medicinal products nor a post-authorization observational study with medicinal products. All patients provided written informed consent before inclusion; those who underwent an additional invasive procedure (colonoscopy or capsule endoscopy) signed a separate, procedure-specific informed consent form prior to the procedure.

### Fecal calprotectin

Fecal calprotectin was quantitatively determined at the HGUGM biochemistry laboratory by chemiluminescent immunoassay (LIAISON Calprotectin Assay, DiaSorin, Saluggia, Italy), with positivity set at ≥50 µg/g according to the manufacturer's specifications and the cutoff commonly used for IBD screening (4). All patients underwent a baseline determination; patients with an initial value <50 µg/g were reassessed at 6–12 months, while those with an initial value ≥50 µg/g were reassessed at 3 months.

Based on the evolution of both determinations, four clinical-protocol subgroups were defined: A, both determinations <50 µg/g; B, first determination <50 µg/g and second ≥50 µg/g; C, first determination ≥50 µg/g and second <50 µg/g; and D, persistent elevation, defined as both determinations ≥50 µg/g, or marked baseline elevation, defined as a first measurement ≥250 µg/g.

A calprotectin delta was additionally defined as the absolute change between the second and first determinations (calprotectin 2 − calprotectin 1, in µg/g), as a continuous measure complementary to, rather than a replacement for, the subgroup classification.

### Endoscopic evaluation and primary outcome

Patients in subgroups B and D, that is, those with a positive result at reassessment or a direct referral indication, underwent colonoscopy. In cases where findings such as diverticula, polyps or hemorrhoids which could potentially explain the elevated calprotectin levels were identified, it was decided to continue with clinical follow-up. However, if the colonoscopy was normal, a capsule endoscopy was recommended.

Colonoscopies were performed after bowel preparation according to the standard clinical protocol of the Endoscopy Unit. Polyethylene glycol- and sodium picosulfate-based regimens were used, with the choice of preparation and dosing schedule left to the discretion of the requesting physician and tailored to individual patient characteristics in accordance with current evidence-based recommendations. All procedures were performed with Olympus 190-series video colonoscopes (PCF-H190) connected to EVIS EXERA III processors (Olympus Medical Systems Corp., Tokyo, Japan).

Small-bowel capsule endoscopy was performed using the PillCam™ SB3 system (Given Imaging Ltd., Medtronic, Yoqneam, Israel). Preparation consisted of dietary measures alone, without the administration of purgatives: patients followed a clear-liquid diet the day before the procedure and fasted for at least 12 h prior to capsule ingestion. Recordings were reviewed with PillCam™ Software version 9.0 by two independent, experienced gastroenterologists blinded to the fecal calprotectin results; discordant cases were adjudicated by a third independent reader. Small-bowel cleansing quality and the small-bowel completion rate, defined as visualization of the cecum before battery depletion, were recorded for all examinations.

Inflammatory activity on capsule endoscopy was quantified using the Lewis score, calculated with the PillCam™ software after reader annotation of villous edema, ulcers/erosions, and stenosis. Lewis score values were interpreted according to established thresholds: <135 as normal or clinically insignificant inflammation, 135–790 as mild inflammatory activity, and ≥790 as moderate-to-severe inflammatory activity. The Lewis score was used as a standardized descriptor of small-bowel inflammatory burden and was interpreted together with the endoscopic pattern and histological findings when available.

Ileocolonoscopy and small-bowel capsule endoscopy assessed all bowel segments accessible to each technique. The primary outcome was subclinical inflammatory bowel disease, defined as endoscopic and/or histological findings suggestive of IBD in any segment examined, identified in a patient without digestive symptoms. Suggestive findings included mucosal breaks (erosions or ulcers), inflammatory mucosal changes (erythema, edema, or friability), and stenosis or resistance at the ileocecal valve. In patients evaluated by ileocolonoscopy, biopsies were obtained whenever technically feasible; histological findings were used to support, but were not required for, the endoscopic diagnosis.

Each patient was classified as having subclinical ileitis, not having subclinical ileitis, or having a non-evaluable digestive outcome. The latter category included patients without endoscopic assessment and without sufficient follow-up data for clinical-protocol subgroup classification; these patients were excluded from diagnostic performance and from analyses of clinical characteristics associated with the outcome. Because all confirmed cases involved the ileum or ileocecal valve, the primary outcome is referred to as subclinical ileitis throughout the manuscript.

### Statistical analysis

Qualitative variables were expressed as absolute frequency and percentage, and quantitative variables as mean ± standard deviation or median (interquartile range, IQR), depending on whether their distribution was compatible with normality, as assessed by the Shapiro–Wilk test or the Kolmogorov–Smirnov test, as appropriate. Two-group comparisons of continuous variables were performed using Welch's *t*-test, which does not assume homogeneity of variances, or the Mann–Whitney *U*-test for non-normally distributed variables; comparisons among more than two groups were performed using one-way ANOVA or the Kruskal–Wallis test. For categorical variables, Pearson's *χ*² test without continuity correction was used when expected frequencies were ≥5 in all cells, Fisher's exact test for 2 × 2 tables with low expected frequencies, and the Fisher–Freeman–Halton exact test for R × C tables with low expected frequencies, calculated by exact enumeration rather than Monte Carlo approximation.

The diagnostic performance of the different screening strategies (sensitivity, specificity, positive predictive value, negative predictive value, and positive/negative likelihood ratios) was estimated together with their exact confidence intervals, using the Clopper–Pearson method (25) and the logarithmic method of Simel et al. (26). Receiver operating characteristic (ROC) curves were constructed for hypothesis-generating purposes, rather than as definitive validation of a new cutoff; the optimal cutoff for each continuous variable was determined using the Youden index (J = sensitivity + specificity − 1), selecting the value that maximizes this index. The confidence interval for the area under the curve (AUC), equivalent to the c-statistic, was estimated using the exact analytical method of DeLong et al. (27), and was compared, as an additional internal validation step, with a bootstrap resampling-based estimate, yielding concordant results between both methods.

Given the small number of ileitis events (*n* = 5) and the consequent risk of quasi-complete separation, a conventional multivariable logistic regression model was not considered appropriate as the primary analysis.

A *p*-value <0.05, two-tailed, was considered statistically significant. The overall statistical analysis (descriptive statistics and between-group comparison tests) was performed with the assistance of an artificial intelligence model (ChatGPT Plus, OpenAI), subsequently confirmed through syntax executed in IBM SPSS Statistics, version 31.0.0 (IBM Corp., Armonk, NY, USA), and validated by a statistician. Exact estimates of diagnostic performance (Clopper-Pearson, Simel et al., and DeLong et al. confidence intervals, and the Youden index) were calculated with the assistance of an artificial intelligence model (Claude Opus 5, Anthropic) using a Python script (SciPy), and were independently verified by the statistician using Epidat software (version 4.2, Xunta de Galicia/Pan American Health Organization–WHO).

## Results

### Baseline characteristics of the study population

Of 73 patients initially assessed, 4 were excluded (1 due to pregnancy and 3 because a baseline fecal calprotectin determination was not available), yielding a final analytic cohort of 69 patients with idiopathic uveitis or scleritis (Figure 1).

**Figure 1:**
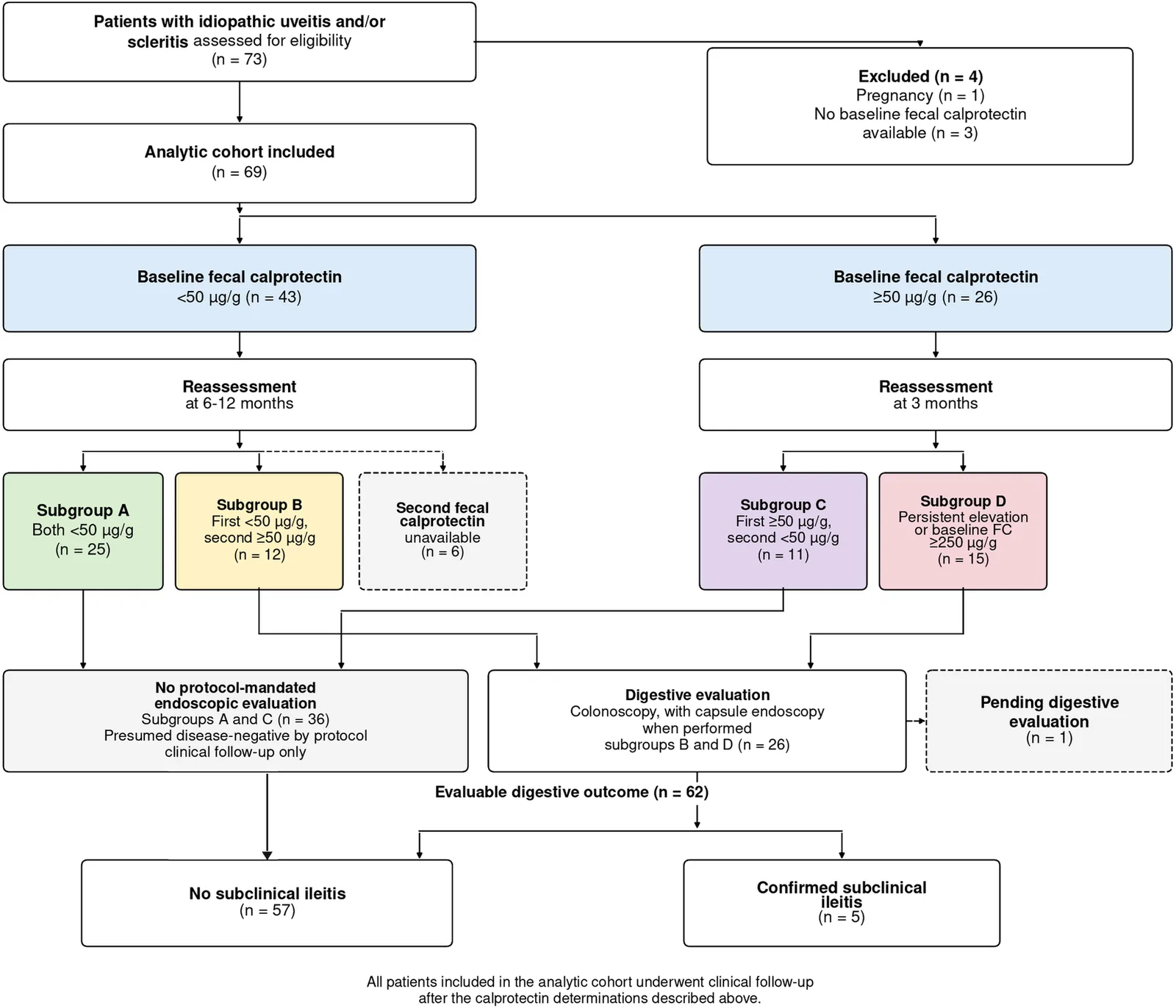
Patient flow diagram. Of 73 patients with idiopathic uveitis and/or scleritis assessed for eligibility, 4 were excluded (1 due to pregnancy and 3 due to unavailable baseline fecal calprotectin), yielding an analytic cohort of 69 patients. Patients with baseline fecal calprotectin (FC) < 50 µg/g (*n* = 43) were reassessed at 6–12 months, whereas those with baseline FC ≥50 µg/g (*n* = 26) were reassessed at 3 months. Based on FC evolution, patients were classified into subgroup A (both determinations <50 µg/g, *n* = 25), subgroup B (first <50 µg/g, second ≥50 µg/g, *n* = 12), subgroup C (first ≥50 µg/g, second <50 µg/g, *n* = 11), or subgroup D (persistent elevation or baseline FC ≥250 µg/g, *n* = 15); 6 patients remained non-classifiable because the second FC determination was unavailable. Patients in subgroups A and C (*n* = 36) did not undergo protocol-mandated endoscopic evaluation and were presumed disease-negative by protocol, with clinical follow-up only. Patients in subgroups B and D were assigned to digestive evaluation; 26 underwent digestive evaluation and 1 was pending. Overall, 62 patients had an evaluable digestive outcome: no subclinical ileitis in 57 and confirmed subclinical ileitis in 5.

The mean age of the population was 46.4 ± 15.9 years, with a sex distribution of 38 women (55.1%) and 31 men (44.9%). Overall, 23.2% of patients were current smokers and 2.9% former smokers; 13.0% had another concomitant immune-mediated disease, none of which was considered associated with or causative of the ocular inflammation, and 52.2% were receiving active immunosuppressive treatment (synthetic disease-modifying antirheumatic drugs in 36.2% and biologic agents in 18.8%). Regarding ocular phenotype, anterior uveitis was the most frequent anatomical form (36.2%), followed by scleritis (20.3%; all anterior except two cases of posterior scleritis), intermediate uveitis (15.9%), posterior uveitis (14.5%), and panuveitis (13.0%); 76.8% of patients had active ocular inflammation at the time of inclusion. Anterior and posterior segment complications were recorded in 37.7% and 42.0% of patients, respectively, and median best-corrected visual acuity in the worse eye was 0.10 (0.00–0.30) logMAR.

Median baseline fecal calprotectin was 36.00 (IQR: 8.66–74.80) µg/g, with a second determination available in 62 of the 69 patients (median 33.10 µg/g, IQR 12.25–93.90). The remaining baseline characteristics of the cohort are detailed in Table 1, with additional demographic, laboratory and ocular variables provided in Supplementary Table 1.

### Fecal calprotectin evolution and clinical-protocol subgroups

Of the 69 patients in the cohort, 63 could be classified into one of the four clinical-protocol subgroups based on the evolution of their two fecal calprotectin determinations, whereas 6 remained non-classifiable because the scheduled second determination was pending. The distribution was as follows: subgroup A (both determinations <50 µg/g), 25 patients; subgroup B (first <50, second ≥50 µg/g), 12 patients; subgroup C (first ≥50, second <50 µg/g), 11 patients; and subgroup D (persistent elevation or baseline fecal calprotectin ≥250 µg/g), 15 patients. Within subgroup D, 14 of the 15 patients met this classification through persistent elevation of fecal calprotectin at both determinations, 5 of whom had confirmed subclinical ileitis. The remaining patient was classified after a single baseline fecal calprotectin determination ≥250 µg/g and did not have confirmed ileitis.

When patients were instead grouped according to their baseline fecal calprotectin category (<50 vs. ≥50 µg/g), the second determination remained significantly higher in the ≥50 µg/g group (median 88.80 vs. 18.60 µg/g; *p* = 0.008) (Table 2); the corresponding comparison for baseline fecal calprotectin itself is not shown, as it would be circular with the very definition of the two groups.

**Table 2:** Comparative analysis of baseline characteristics by baseline fecal calprotectin group (<50 vs. ≥50 µg/g).

Variable	<50 µg/g (*n* = 43)	≥50 µg/g (*n* = 26)	*p*-value
Age, years	44.9 ± 16.6	48.8 ± 14.7	0.316
Sex, male	21 (48.8%)	10 (38.5%)	0.460
Smoking status, current/former/never	10 (23.3%)/1 (2.3%)/32 (74.4%)	6 (23.1%)/1 (3.8%)/19 (73.1%)	1.000
Systemic corticosteroid therapy (oral prednisone and/or methylprednisolone pulses), yes	18 (41.9%)	8 (30.8%)	0.445
Conventional or biologic DMARD therapy^a^, yes	22 (51.2%)	8 (30.8%)	0.134
Active immunosuppression, yes	26 (60.5%)	10 (38.5%)	0.088
Fecal calprotectin, 2nd determination, µg/g	18.60 (10.00–52.80)	88.80 (24.70–107.00)	0.008*
Type of ocular inflammation (Anterior/Intermediate/Posterior/Panuveitis/Scleritis)	14 (32.6%)/9 (20.9%)/6 (14%)/4 (9.3%)/10 (23.3%)	11 (42.3%)/2 (7.7%)/4 (15.4%)/5 (19.2%)/4 (15.4%)	0.432
Ocular laterality (Unilateral/Bilateral)	19 (44.2%)/24 (55.8%)	12 (46.2%)/14 (53.8%)	1.000
Active ocular disease at inclusion^d^, yes	33 (76.7%)	20 (76.9%)	1.000
Anterior segment complications, yes	14 (32.6%)	12 (46.2%)	0.310
Posterior segment complications, yes	17 (39.5%)	12 (46.2%)	0.623
Macular thickness, affected eye, µm	263.00 (250.00–290.00)	265.50 (256.00–279.00)	0.879
RNFL^b^ thickness, affected eye, µm	109.50 (106.00–118.25)	107.00 (105.00–119.00)	0.331
Duration of ocular disease, months	27 (8–47)	88 (16–126)	0.014*
BCVA^c^ LogMAR, worse eye	0.05 (0.00–0.15)	0.22 (0.00–0.30)	0.051
Prolonged ocular disease course (≥60 months), yes	6/42 (14.3%)	16/25 (64.0%)	<0.001*

DMARD, disease-modifying antirheumatic drug; RNFL, retinal nerve fiber layer; BCVA, best-corrected visual acuity. Quantitative variables are compared using Welch's *t*-test or the Mann–Whitney *U*-test, depending on their distribution; qualitative variables, using Pearson's *χ*² test, Fisher's exact test (2 × 2 tables), or the Fisher–Freeman–Halton exact test (R × C tables), calculated by exact enumeration, not Monte Carlo approximation. *p* < 0.05 considered statistically significant (bold).

a Synthetic and biologic DMARD categories are not mutually exclusive. Expressed as overall exposure (yes/no); no individual drug-level breakdown is available.

b Retinal nerve fiber layer thickness, obtained using the “3D Disc-RNFL Total Thickness” scan protocol (Topcon 3D OCT-1 Maestro).

c Visual acuity converted to its LogMAR equivalent from the original decimal recording.

d Percentages for prolonged ocular disease course were calculated among patients with available duration data: 42 in the <50 µg/g group and 25 in the ≥50 µg/g group.

None of the general demographic or clinical variables showed significant differences between the two groups, including age, sex, smoking status, systemic corticosteroid therapy (oral prednisone and/or methylprednisolone pulses), conventional or biologic DMARD therapy, or overall active immunosuppression (all *p* > 0.05) (Table 2). Likewise, no differences were observed in the Charlson comorbidity index, the presence of another immune-mediated disease, NSAID or PPI use, general laboratory parameters (hemoglobin, leukocytes, ESR, rheumatoid factor), or intraocular pressure (all *p* > 0.05) (Supplementary Table 2). Most ophthalmologic variables were also similar between groups, including type and laterality of ocular inflammation, ocular activityᵈ, anterior or posterior segment complications, and macular or RNFL thickness (all *p* > 0.05) (Table 2).

Two ophthalmologic variables did show significant differences between groups.

Duration of ocular disease was significantly longer in the ≥50 μg/g group (median 88 vs. 27 months; *p* = 0.014). Likewise, a prolonged ocular disease course, defined as a duration of ≥60 months, was considerably more frequent in the ≥50 μg/g group (64%) than in the <50 μg/g group (14.3%; *p* < 0.001), a pattern consistent with the difference observed in disease duration itself (Table 2); both comparisons are restricted to the *n* = 25 and *n* = 42 patients, respectively, with available duration data. Best-corrected visual acuity in the worse eye did not differ significantly between groups (median logMAR 0.22 vs. 0.05; *p* = 0.051).

### Diagnostic performance of fecal calprotectin for the detection of subclinical inflammatory bowel disease

The diagnostic performance of five fecal calprotectin-based screening strategies for the detection of subclinical ileitis was assessed (Table 3). Because only patients in subgroups B and D underwent endoscopic verification, while those in subgroups A and C were presumed disease-negative by protocol, these estimates are subject to partial verification bias and should be interpreted accordingly (see Limitations). All single-cutoff strategies, except the calprotectin increase strategy, achieved 100% sensitivity (95% CI: 47.8–100.0), with no false negatives observed among protocol-verified patients undergoing endoscopy. Specificity increased progressively from isolated baseline fecal calprotectin (63.2%; 95% CI: 49.3–75.6) to the clinical-protocol subgroup D (82.5%; 95% CI: 70.1–91.3) and the strict subgroup D (84.2%; 95% CI: 72.1–92.5), with a corresponding increase in the positive likelihood ratio (LR+) from 2.71 (95% CI: 1.93–3.81) to 5.70 (95% CI: 3.25–10.01) and 6.33 (95% CI: 3.48–11.53), respectively. According to standard likelihood ratio magnitude rules, these LR + values fall within the small-to-moderate range of change in post-test probability. The strategy based solely on a calprotectin increase ≥50 µg/g showed, in contrast, lower sensitivity (40.0%; 95% CI: 5.3–85.3) but higher specificity (85.7%; 95% CI: 73.8–93.6).

**Table 3:** Diagnostic performance of fecal calprotectin-based screening strategies for the detection of subclinical ileitis.

Parameter	Baseline FC ≥50	2nd FC ≥50	Subgroup D	Strict subgroup D	FC increase ≥50
*n* ^c^	62	61	62	62	61
Se (95% CI^a^)	100.0% (47.8–100.0)	100.0% (47.8–100.0)	100.0% (47.8–100.0)	100.0% (47.8–100.0)	40.0% (5.3–85.3)
Sp (95% CI^a^)	63.2% (49.3–75.6)	67.9% (54.0–79.7)	82.5% (70.1–91.3)	84.2% (72.1–92.5)	85.7% (73.8–93.6)
PPV (95% CI^a^)	19.2% (6.6–39.4)	21.7% (7.5–43.7)	33.3% (11.8–61.6)	35.7% (12.8–64.9)	20.0% (2.5–55.6)
NPV (95% CI^a^)	100.0% (90.3–100.0)	100.0% (90.7–100.0)	100.0% (92.5–100.0)	100.0% (92.6–100.0)	94.1% (83.8–98.8)
LR+ (95% CI^b^)	2.71 (1.93–3.81)	3.11 (2.13–4.55)	5.70 (3.25–10.01)	6.33 (3.48–11.53)	2.80 (0.80–9.78)
LR− (95% CI^b^)	0.00 (NC)	0.00 (NC)	0.00 (NC)	0.00 (NC)	0.70 (0.34–1.44)
Accuracy	66.1%	70.5%	83.9%	85.5%	82.0%

Se, sensitivity; Sp, specificity; PPV, positive predictive value; NPV, negative predictive value; LR+/LR−, positive/negative likelihood ratio; NC, not calculable (division by zero when F*P* = 0 or TN = 0); FC, fecal calprotectin.

a Exact Clopper–Pearson confidence interval.

b Confidence interval by the logarithmic method of Simel, Samsa, and Matchar.

c Negative ileitis status was presumed rather than endoscopically confirmed in patients from subgroups A and C, who did not undergo colonoscopy or capsule endoscopy per protocol (only 26 of 69 patients, corresponding to subgroups B and D, underwent endoscopic evaluation); see Limitations for a discussion of the resulting partial verification bias.

d Strict subgroup D comprised the 14 of 15 subgroup D patients in whom persistent fecal calprotectin elevation was confirmed at both determinations; it excluded the one patient classified into subgroup D solely on the basis of baseline fecal calprotectin ≥250 µg/g.

ROC curve analyses (Table 4; Figure 2) showed an area under the curve (AUC) of 0.809 (DeLong 95% CI: 0.652–0.966) for baseline fecal calprotectin and 0.932 (DeLong 95% CI: 0.850–1.000) for the second determination, with optimal Youden index cutoffs of 58 µg/g and 92.1 µg/g, respectively. As a formal comparison beyond these individual confidence intervals, the ROC curves of the baseline and second determinations were compared using the DeLong test for correlated ROC curves, with no statistically significant difference found in their c-statistics (AUC) (*n* = 61, *p* = 0.242), likely due to the small number of ileitis cases (*n* = 5). Because the paired DeLong comparison between baseline and second-determination fecal calprotectin AUCs did not reach statistical significance (*n* = 61, *p* = 0.242), the numerically higher AUC of the second determination should be interpreted as exploratory and does not demonstrate statistical superiority of serial over single-timepoint fecal calprotectin measurement. The fecal calprotectin delta showed moderate discriminative performance (AUC: 0.704). Duration of ocular disease also showed good discriminative performance for subclinical ileitis (AUC: 0.879), although this finding should be interpreted with caution because it was based on only five ileitis cases and was not a predefined screening marker. Visual acuity in the worse eye showed no meaningful discriminative performance (AUC: 0.504; DeLong 95% CI: 0.248–0.759), and these data do not support its use as a screening marker in this cohort.

**Table 4:** Exploratory ROC curves: area under the curve (AUC) with DeLong confidence interval and optimal cutoff according to the Youden index.

Variable	n	Events	AUC	DeLong 95% CI^a^	Bootstrap 95% CI^b^	Cutoff (Youden)	Se/Sp at cutoff
Baseline calprotectin	62	5	0.809	0.652–0.966	0.659–0.969	58 µg/g	100.0%/64.9%
Second calprotectin	61	5	0.932	0.850–1.000	0.846–1.000	92.1 µg/g	100.0%/80.4%
Calprotectin delta	61	5	0.704	0.356–1.000	0.329–1.000	8 µg/g	80.0%/69.6%
Duration of ocular disease	61	5	0.879	0.774–0.983	0.754–0.970	74 months	100.0%/73.2%
BCVA LogMAR, worse eye	62	5	0.504	0.248–0.759	0.217–0.767	0.155	60.0%/56.1%

BCVA, best-corrected visual acuity. With only 5 ileitis events, both confidence interval methods are asymptotic approximations of limited precision; the resulting intervals are accordingly wide and should be interpreted with caution.

a Exact analytical method of DeLong et al.

b Bootstrap resampling-based estimate, presented as an additional internal validation (see Methods).

**Figure 2:**
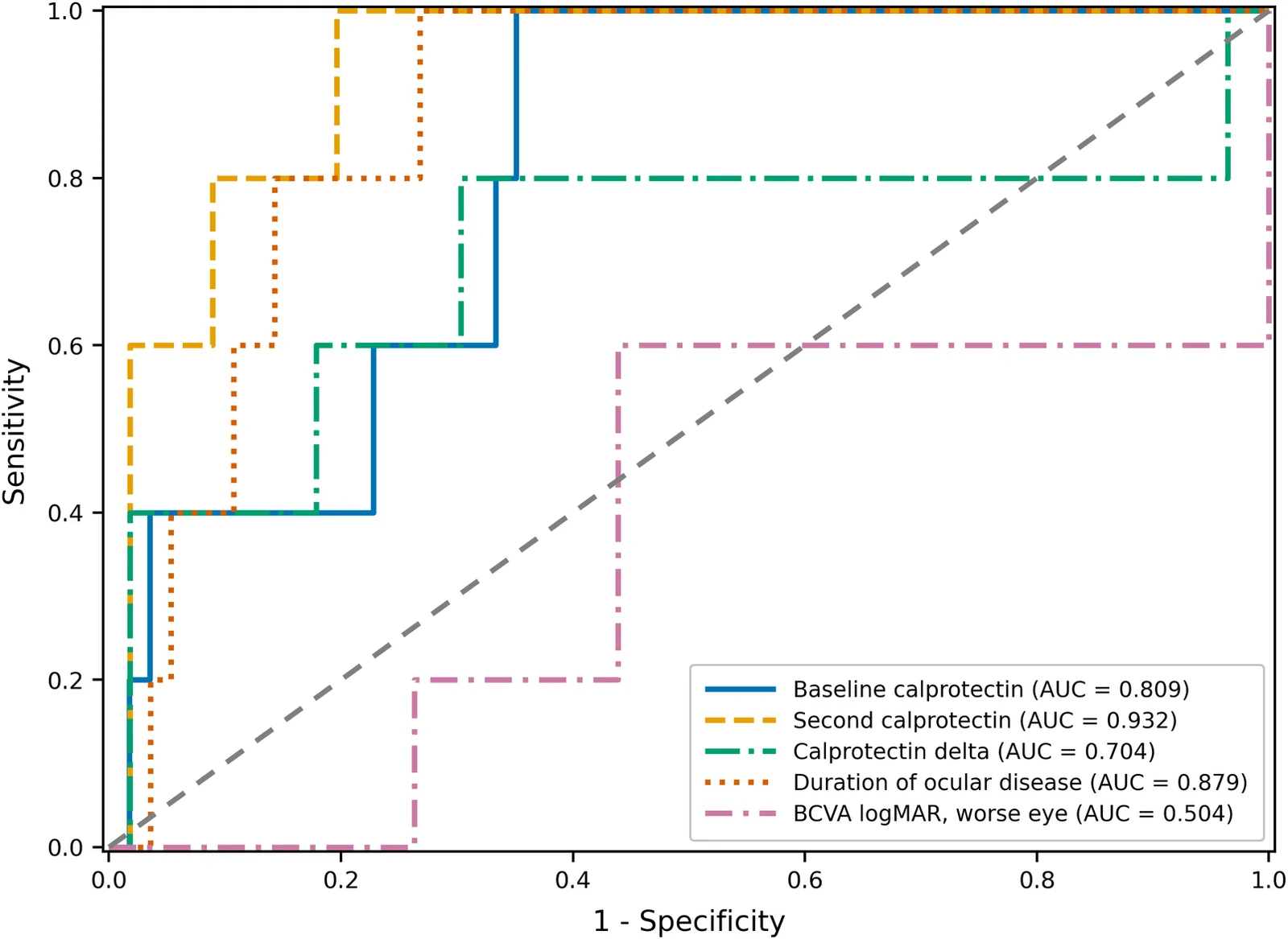
Exploratory receiver operating characteristic (ROC) curves for the detection of subclinical ileitis. ROC curves are shown for baseline fecal calprotectin, the second fecal calprotectin determination, the calprotectin delta, the duration of ocular disease, and best-corrected visual acuity (BCVA) LogMAR in the worse eye, together with their corresponding area under the curve (AUC). The grey diagonal reference line indicates no discriminative ability (AUC = 0.5). ROC, receiver operating characteristic; AUC, area under the curve; BCVA, best-corrected visual acuity.

### Clinical characteristics associated with subclinical inflammatory bowel disease

Of the 69 patients included, 62 had a properly established digestive outcome. Seven patients were excluded from the outcome analysis: 6 because they could not be classified into the clinical-protocol subgroups (no second fecal calprotectin determination available and baseline fecal calprotectin <250 µg/g), and 1 because the endoscopic evaluation was pending. Within this restricted, evaluable cohort, subclinical ileitis was confirmed in 5 patients (8.1%) and ruled out in 57 (91.9%) (Table 5). All five confirmed cases involved the distal small bowel: the terminal ileum in 4 patients and the ileocecal valve in 1. Table 6 details the endoscopic, histological, symptomatic, and therapeutic data for each of the five cases.

**Table 5:** Comparative analysis by presence or absence of confirmed subclinical ileitis.

Variable	Ileitis (*n* = 5)	No ileitis (*n* = 57)	*p*-value
Age, years	54.4 ± 13.2	46.3 ± 16.2	0.251
Sex, male/female	3 (60.0%)/2 (40.0%)	22 (38.6%)/35 (61.4%)	0.385
Smoking status, current/former/never	1 (20.0%)/0 (0.0%)/4 (80.0%)	14 (24.6%)/2 (3.5%)/41 (71.9%)	1.000
Oral prednisone, none/<5/5–10/>10 mg/day	5 (100.0%)/0/0/0	35 (61.4%)/4 (7.0%)/6 (10.5%)/12 (21.1%)	0.721
Methylprednisolone pulses, none/125/250/500 mg × 3	5 (100.0%)/0/0/0	43 (75.4%)/2 (3.5%)/10 (17.5%)/2 (3.5%)	0.699
Synthetic DMARDs^a^, yes	0 (0.0%)	24 (42.1%)	0.147
Biologic DMARDs^a^, yes	0 (0.0%)	12 (21.1%)	0.573
Active immunosuppression, yes	0 (0.0%)	32 (56.1%)	0.022*
Baseline fecal calprotectin, µg/g	84.10 (62.70–193.00)	37.00 (10.00–74.80)	0.024*
Fecal calprotectin, 2nd determination, µg/g	220.00 (107.00–427.00)	24.45 (11.50–55.23)	0.002*
Type of ocular inflammation	Anterior 4 (80.0%); Posterior 1 (20.0%)	Anterior 18 (31.6%); Intermediate 8 (14.0%); Posterior 8 (14.0%); Panuveitis 9 (15.8%); Scleritis 14 (24.6%)	0.268
Ocular laterality (Unilateral/Bilateral)	Unilateral 2 (40.0%); Bilateral 3 (60.0%)	Unilateral 26 (45.6%); Bilateral 31 (54.4%)	1.000
Active ocular disease at inclusion^d^, yes	0 (0.0%)	25 (43.9%)	0.146
IOP, mmHg	12.00 (12.00–13.00)	15.00 (12.00–16.00)	0.041
Macular thickness, affected eye, µm	260.00 (233.00–275.00)	267.00 (253.75–284.50)	0.348
RNFL^b^ thickness, affected eye, µm	106.00 (101.00–107.00)	108.00 (105.00–118.00)	0.291
Duration of ocular disease, months	126 (110–140)	38 (7–75)	0.006*
BCVA^c^ LogMAR, worse eye	0.15 (0.00–0.22)	0.10 (0.00–0.30)	0.989
Clinical-protocol subgroup (A/B/C/D)	A 0; B 0; C 0; D 5 (100.0%)	A 25 (43.9%); B 11 (19.3%); C 11 (19.3%); D 10 (17.5%)	<0.001*

DMARD, disease-modifying antirheumatic drug; IOP, intraocular pressure; RNFL, retinal nerve fiber layer; BCVA, best-corrected visual acuity. Seven patients are excluded from this analysis: six because they could not be classified into the clinical-protocol subgroups, and one because the endoscopic evaluation was pending (see Methods). Quantitative variables are compared using Welch's *t*-test or the Mann–Whitney *U*-test, depending on their distribution; qualitative variables, using Fisher's exact test or the Fisher–Freeman–Halton exact test (calculated by exact enumeration, not Monte Carlo approximation). *p* < 0.05 considered statistically significant (bold).

a Expressed as overall exposure (yes/no); no individual drug-level breakdown is available.

b Retinal nerve fiber layer thickness, obtained using the “3D Disc-RNFL Total Thickness” scan protocol (Topcon 3D OCT-1 Maestro).

c Visual acuity converted to its LogMAR equivalent.

d Active ocular disease at inclusion was dichotomized from the clinical status recorded at inclusion: patients classified as active with treatment or active without treatment were considered active, whereas those classified as inactive with treatment or inactive without treatment were considered inactive.

Charlson comorbidity index, presence of another immune-mediated disease, NSAID and PPI use, general laboratory parameters (hemoglobin, leukocytes, ESR, rheumatoid factor), and anterior/posterior segment complications are presented in Supplementary Table 4.

**Table 6:** Individual clinical, biomarker, endoscopic, and follow-up characteristics of the five patients with confirmed subclinical ileitis.

Variable	Case 1	Case 2	Case 3	Case 4	Case 5
Ocular and biomarker profile					
Age range, years	50–59	50–59	60–69	30–39	60–69
Sex	Male	Female	Female	Male	Male
Ocular diagnosis	Bilateral anterior uveitis	Bilateral anterior uveitis	Unilateral anterior uveitis	Unilateral anterior uveitis	Bilateral posterior uveitis
Ocular disease duration, months	152	126	140	110	74
SUN uveitis course	Chronic	Persistent	Persistent	Recurrent	Chronic
HLA-B27	Negative	Negative	Negative	Positive	Negative
NSAID/PPI use	No/No	No/No	No/No	No/No	No/Yes*
Immunosuppressive treatment at inclusion	No	No	No	No	No
Baseline/second FC, µg/g	58.0/427.0	62.7/451.0	193.0/220.0	84.1/92.1	239.0/107.0
Digestive evaluation					
Diagnostic modality	Capsule endoscopy after normal ileocolonoscopy	Ileocolonoscopy	Ileocolonoscopy; ileal intubation not achieved	Capsule endoscopy after normal ileocolonoscopy	Ileocolonoscopy
Endoscopic findings	Three fibrin-covered erosions (1–2 mm) with an erythematous halo in the terminal ileum, unchanged from a previous capsule study; suggestive of Crohn's disease	Inflammatory-appearing terminal ileitis; internal hemorrhoids	Increased resistance at the ileocecal valve precluding ileoscopy, considered suggestive of inflammation at that level; normal colonic mucosa	Isolated superficial ulcer of the preterminal ileum on the first capsule study; two fibrin-covered ulcers (3 and 8 mm) with an erythematous halo in the terminal ileum on the second; suggestive of Crohn's disease	Two superficial longitudinal erosions of the terminal ileum, consistent with nonspecific ileitis; colonic polyps and uncomplicated diverticulosis
Histology	Not obtained	Ileal and colonic mucosa without significant abnormalities	Ileocecal valve: mild acute colitis, potentially compatible with inflammatory bowel disease	Not obtained	Ileal mucosa with lamina propria edema and mild focal nonspecific inflammation
Lewis score	225	N/A (ileocolonoscopy)	N/A (ileocolonoscopy)	233	N/A (ileocolonoscopy)
Follow-up and current status					
Digestive symptoms at 12/24 months	No/No	Yes/Yes	No/Yes	No/No	No/No
Treatment change prompted by the finding	No	Yes (adalimumab)	No	No	No
Current status at longest follow-up	Resolved on subsequent evaluation; in follow-up	Ileal Crohn's disease; Axial spondyloarthritis	Suspected Crohn's disease, unconfirmed	Suspected Crohn's disease, unconfirmed	Resolved on subsequent evaluation; in follow-up

FC, fecal calprotectin; HLA-B27, human leukocyte antigen B27; NSAID, non-steroidal anti-inflammatory drug; PPI, proton-pump inhibitor; N/A, not applicable or not available, according to context.

All five patients were classified within clinical-protocol subgroup D; none received active immunosuppression or NSAIDs at inclusion, and only Case 5 received a PPI.

Lewis score is reported only for capsule endoscopy studies as first-capsule; second-capsule values. Capsule endoscopy does not permit tissue sampling. Age is reported in ranges to preserve confidentiality.

* PPI: omeprazole 20 mg/day.

During follow-up, two of the five patients developed digestive symptoms (at 12 and at 24 months, respectively), and in one of them the finding prompted initiation of anti-TNF therapy. In the remaining three, the diagnosis remained subclinical throughout follow-up.

The clinical course of the five confirmed cases of subclinical ileitis during follow-up is summarized in Table 6. At the most recent available follow-up, one patient (Case 2) had received a formal diagnosis of ileal Crohn's disease together with HLA-B27-negative axial spondyloarthritis. Two patients (Cases 3 and 4) remained under evaluation for suspected Crohn's disease, without a definitive diagnosis at the time of this analysis. The remaining two patients (Cases 1 and 5) had shown resolution of the initial endoscopic findings on subsequent evaluation and remained under clinical follow-up without ongoing gastrointestinal symptoms.

Two cases were identified by capsule endoscopy and three by ileocolonoscopy. Endoscopic findings comprised ulcers, fibrin-covered erosions, and inflammatory or nonspecific ileal changes, together with resistance at the ileocecal valve in one patient. Biopsies were obtained in the three patients evaluated by ileocolonoscopy and showed inflammatory changes in 2 of them; capsule endoscopy does not permit tissue sampling (Table 6).

One additional patient, classified in clinical-protocol subgroup C (elevated baseline fecal calprotectin with normalization at reassessment), developed digestive symptoms during follow-up and was subsequently diagnosed with ulcerative colitis through the standard clinical pathway, outside the study's biomarker-guided endoscopic algorithm. Because this patient did not undergo protocol-defined endoscopic evaluation at the time of calprotectin normalization, this case was not counted among the five confirmed subclinical ileitis outcomes; however, it represents a false negative of the calprotectin-normalization-based decision rule within subgroup C, and it constrains any claim that the negative predictive value of a normalized second determination is 100% at the level of the overall screening strategy, as opposed to the specific subgroup D pathway with endoscopic verification. Patients with a normalized calprotectin trajectory who subsequently develop digestive symptoms should be clinically reassessed regardless of their biomarker-based classification.

HLA-B27 was positive in 1 of the 5 patients with confirmed subclinical ileitis (Case 4) and negative in the remaining four (Table 6). None of the five patients was taking nonsteroidal anti-inflammatory drugs, either chronically or at the time of fecal calprotectin sampling. One patient (Case 5) was taking a proton pump inhibitor at a dose of 20 mg/day on a chronic and continuous basis.

The absence of active immunosuppressive treatment was significantly associated with the presence of subclinical ileitis: none of the 5 patients with confirmed ileitis were receiving active immunosuppression, compared with 56.1% of patients without ileitis (*p* = 0.022). As expected, both baseline fecal calprotectin (median 84.10 vs. 37.00 µg/g; *p* = 0.024) and the second determination (median 220.00 vs. 24.45 µg/g; *p* = 0.002) were significantly higher in patients with ileitis. All ileitis cases were concentrated in subgroup D (100%; *p* < 0.001), with no cases in subgroups A, B, or C. In the evaluable cohort, intraocular pressure was lower in patients with subclinical ileitis than in those without ileitis (12.00 vs. 15.00 mmHg; *p* = 0.041). Given the small number of confirmed ileitis cases (*n* = 5) and the exploratory nature of this comparison, this finding should be interpreted with caution and requires confirmation in larger cohorts.

No significant differences were observed between patients with and without ileitis in age, sex, smoking status, comorbidity, other immune-mediated disease, any other treatment variable (NSAIDs, PPIs, oral corticosteroids, methylprednisolone pulses, synthetic DMARDs, or biologic DMARDs considered individually), general laboratory parameters (hemoglobin, leukocytes, ESR, rheumatoid factor), type or laterality of ocular inflammation, ocular activityᵈ, ocular complications, cellularity, macular or RNFL thickness, or visual acuity (all *p* > 0.05). The duration of ocular disease was significantly longer in patients with ileitis (median 126 vs. 38 months; *p* = 0.006), although this finding should be interpreted in the context of the small number of confirmed ileitis cases (Supplementary Table 4).

## Discussion

### Principal findings

In this prospective cohort of 69 patients with idiopathic uveitis or scleritis, serial, rather than isolated, fecal calprotectin determinations identified subclinical ileitis, confirmed endoscopically, in 5 of 62 evaluable patients (8.1%), all within subgroup D, defined by persistent biomarker elevation or a single baseline fecal calprotectin determination ≥250 μg/g. This finding supports the study hypothesis: fecal calprotectin, interpreted dynamically rather than as a single measurement, is a useful tool for unmasking subclinical ileitis in patients with no prior digestive suspicion at the time of ophthalmological diagnosis.

These results align with growing evidence that ocular inflammation can precede, rather than merely accompany, inflammatory bowel disease. King et al., in a UK primary-care cohort of over 38,000 patients with ophthalmic disorders vs. 150,000 controls, reported an increased risk of subsequent IBD diagnosis (HR: 2.25, particularly for Crohn's disease), with a mean diagnostic interval exceeding two years (13); this is precisely the delay that ECCO guidelines identify as a priority area for multidisciplinary improvement, since ocular, musculoskeletal, and mucocutaneous manifestations often precede the intestinal diagnosis (8). Hedin et al. attributed this independence to a shared immunological process with its own dynamics, rather than a simple epiphenomenon of intestinal activity (28), consistent with our detection of ileitis in patients without digestive symptoms or suspicion. Our prevalence of subclinical ileitis (8.1% overall and 33.3% within subgroup D) is comparable, in the reverse direction, to the prevalence of ocular extraintestinal manifestations reported in IBD cohorts by Kilic et al. (7) and Li et al. (9), and to the pattern of digestive-activity-independent ocular involvement described by Pytrus et al. and Mintz et al. (10, 11) and by Songel-Sanchis and Cosín-Roger (12), who additionally emphasized the need for active ophthalmological screening in established IBD (12); our study approaches the same gut-eye axis from the opposite direction, from idiopathic uveitis/scleritis toward subclinical ileitis rather than the reverse, offering bidirectional validation of this relationship. This is further supported by Yavari et al., who describe anterior uveitis as the most frequent, and at times earliest, ocular manifestation in HLA-B27–associated disease among more than 22,000 patients (15), and by the bidirectional Mendelian randomization study of Zhong et al., which provides genetic evidence of directional causality from IBD susceptibility to uveitis (OR: 1.14), without evidence of reverse causation (14).

The most direct methodological precedent for our protocol is Klingberg et al.'s longitudinal study in ankylosing spondylitis, an HLA-B27–related entity, as reviewed by Bittar and Deodhar (29), which used serial fecal calprotectin to predict IBD development and found this association specifically with anti-TNF, but not recombinant fusion-protein, therapy (19); this precedent both validates the longitudinal biomarker approach and anticipates the pharmacological nuance we revisit below regarding immunosuppression in our own cohort.

The low prevalence of HLA-B27 in our cohort should be interpreted in the context of its specific source population, which consisted of patients with recurrent, chronic, or persistent idiopathic uveitis or scleritis and was not enriched for spondyloarthritis. By contrast, spondyloarthritis-focused cohorts such as EISER have reported a higher frequency of undiagnosed IBD, particularly in patients with axial disease, with most cases corresponding to ileal or ileocolonic Crohn's disease (30).

### Mechanistic basis of the gut-eye axis

Four complementary mechanisms outline the pathophysiological basis of the gut-eye axis.

First, intestinal barrier dysfunction and dysbiosis allow bacterial antigens to translocate and secondarily compromise the blood-ocular barrier (28, 31), with direct evidence in immune-mediated uveitis (32), microbial metabolites with ocular effects (33), and a resident intraocular microbiota (34). In HLA-B27-associated uveitis specifically, this allele reshapes the gut microbiome toward a proinflammatory microecology (35).

Second, aberrant lymphocyte trafficking between the gut and other immune-privileged tissues offers a cellular mechanism: the “vedolizumab paradox” (36), described in joint disease, is conceptually transferable to uveitis.

Third, calprotectin (S100A8/S100A9) is an innate-immunity alarmin rather than a mere neutrophilia marker (16), signaling through TLR4/RAGE and directly reflecting intraocular inflammation in uveitis (17, 18).

Fourth, genetic and epigenetic susceptibility is shared: HLA-B27 and IL23R loci overlap between uveitis and spondyloarthritis-associated IBD (29, 35, 37), individual genetic heterogeneity is confirmed by whole-exome sequencing (38, 39), and Mendelian randomization supports causality (14). An epigenetic component (40) may explain why only some genetically predisposed patients develop overt intestinal disease, a variability that polygenic risk scores may eventually help stratify, despite current barriers to clinical translation (41).

Together, these four mechanisms provide a plausible model for how persistent elevation of a systemic myeloid biomarker such as fecal calprotectin can signal subclinical intestinal disease in a patient whose disease first manifested in the eye.

### The immunosuppression paradox

A specific and seemingly counterintuitive finding is the complete absence of active immunosuppressive treatment in the 5 patients with confirmed ileitis, compared with 56.1% (32/57) of patients without ileitis (*p* = 0.022). This admits two non-exclusive interpretations. Causally, systemic immunosuppression may be suppressing or masking a subclinical intestinal inflammation that would otherwise become biochemically and endoscopically evident. This applies particularly to anti-TNF agents, whose role in jointly controlling ocular and intestinal inflammation is supported by the VISUAL I/II (42, 43) and ADVISE (44) trials.

Three further lines of evidence converge here: Klingberg et al. found that anti-TNF specifically reduced progression to overt IBD in ankylosing spondylitis (19); Bouzid et al. described how systemic treatment modifies the course of HLA-B27–associated uveitis (45); and the FOCUS guidance recommends considering associated systemic disease, including IBD, when selecting therapy (46).

Alternatively, and non-causally, the absence of immunosuppression could simply mark a less globally controlled inflammatory disease in which ocular and intestinal involvement progress in parallel; our observational design cannot distinguish between these hypotheses, and the vedolizumab paradox described by Varkas et al. (36) further cautions that the relationship between systemic immunomodulation and organ-specific inflammation depends on the therapeutic target and trafficking pathway blocked, rather than being uniformly protective. Supporting a genuine biological signal over a treatment-related artifact, both baseline (84.10 vs. 37.00 µg/g; *p* = 0.024) and second-determination (220.00 vs. 24.45 µg/g; *p* = 0.002) calprotectin values were significantly higher in patients with ileitis, in line with the dual systemic/local inflammatory role of calprotectin described by Carnazzo et al. (47).

### Clinical implications for a screening algorithm

These findings suggest that a biomarker-guided screening approach may help identify patients with idiopathic uveitis or scleritis who warrant digestive evaluation, although its clinical utility should be interpreted as exploratory and requires confirmation in larger, independently validated cohorts. The diagnostic performance of clinical-protocol subgroup D (LR+ 5.70 [95% CI: 3.25–10.01]; NPV 100%) corresponds to a moderate shift in post-test probability; however, its negative predictive value must be interpreted with caution given the imprecision inherent to only 5 events and the partial verification bias arising from the biomarker-guided design, in which subgroups A and C were not endoscopically verified. These estimates should not be taken to indicate that invasive digestive evaluation can be safely deferred on the basis of a persistently normal calprotectin trajectory alone, a conclusion that would require prospective validation with systematic endoscopic verification of biomarker-negative patients. This mirrors the established role of fecal calprotectin as a non-invasive screening and monitoring tool in IBD itself (3, 4, 47, 48, 49), transferred here to a less explored setting: screening for subclinical ileitis in a population at risk defined by ocular disease, consistent with prior reports of elevated fecal calprotectin in spondyloarthritis-associated ocular inflammation (20) and in subclinical intestinal involvement in Behçet's disease (21).

Serial fecal calprotectin assessment may provide a pragmatic framework for selecting patients with idiopathic uveitis or scleritis who warrant digestive evaluation. In this protocol, patients with baseline fecal calprotectin <50 µg/g were reassessed at 6–12 months, whereas those with baseline values ≥50 µg/g were reassessed at 3 months. Gastroenterology evaluation was prioritized for patients with new elevation at reassessment, persistent elevation, or a single markedly elevated baseline value (≥250 µg/g). However, this approach should be considered exploratory: one subgroup C patient was later diagnosed with ulcerative colitis through the standard clinical pathway, underscoring that new digestive symptoms should prompt reassessment irrespective of prior fecal calprotectin normalization.

The rationale for this approach is supported by King et al.'s report of a mean two-year interval between ocular manifestation and IBD diagnosis (13), suggesting that ocular inflammation may precede clinically recognized intestinal disease by a substantial period. Earlier identification of subclinical intestinal inflammation may therefore be clinically relevant, particularly in light of the treat-to-target principles emphasized in ECCO guidelines for ulcerative colitis (5) and Crohn's disease (50). However, because the paired DeLong comparison between baseline and second-determination fecal calprotectin AUCs did not reach statistical significance (*p* = 0.242), these data should be considered hypothesis-generating. We therefore frame repeat fecal calprotectin testing as a pragmatic, protocol-driven tool for triaging gastroenterology evaluation, rather than as a formally validated improvement in diagnostic discrimination.

### Strengths and limitations

This study presents methodological strengths worth highlighting alongside its limitations. Among the former, the prospective, protocolized nature of the serial, rather than isolated, fecal calprotectin determination reduces the risk of misclassification due to transient elevations attributable to known confounding factors, and the use of exact, deterministic statistical methods (24) for confidence interval estimation, independently verified by the responsible statistician, lends methodological robustness despite the limited sample size.

However, the study's most important limitation is precisely this small number of primary outcome events (*n* = 5), which yields very wide confidence intervals for virtually all diagnostic performance estimates and precludes, as already noted, the fitting of a conventional multivariable regression model owing to quasi-complete separation of the data. Given this, and consistent with the absence of a formal sample size calculation noted in the Methods, this study should be regarded as exploratory and hypothesis-generating rather than as a definitive validation of a screening algorithm. The single-center setting, a tertiary hospital with a dedicated immune-mediated inflammatory disease unit (CEIMI), may also limit the generalizability of the results to populations with a different referral profile.

Six of the 69 patients (8.7%) could not be classified into subgroups A-D owing to insufficient follow-up or a pending second determination, a rate to be expected in a prospective design with reassessment at up to 12 months. These patients were excluded from the analysis rather than being assumed negative for ileitis by default, since their true digestive outcome was unknown: they neither underwent endoscopic evaluation nor completed the follow-up required for classification. Including them would have diluted the “no ileitis” group with cases of indeterminate status. Reassuringly, these 6 patients did not differ significantly from the rest of the cohort in age, baseline fecal calprotectin, duration of ocular disease, or visual acuity (all *p* > 0.05), providing no indication that non-classification was skewed toward a particular phenotype.

Additionally, the absence of endoscopic verification in patients in subgroups A and C introduces a degree of partial verification bias inherent to biomarker-guided screening protocols, in which only patients with higher pretest probability undergo the reference standard. The non-classifiable patients were excluded from the outcome analyses because they neither underwent endoscopic evaluation nor completed the follow-up required for subgroup classification. This limitation, shared with prior fecal calprotectin screening literature (51), means that the true specificity and negative predictive value could be marginally overestimated if any undetected ileitis were present among the presumed-negative patients. Consistent with this limitation, one patient classified in subgroup C developed digestive symptoms during follow-up and was subsequently diagnosed with ulcerative colitis through the standard clinical pathway; because this patient became symptomatic and was diagnosed outside the study's biomarker-guided algorithm, the case did not meet the definition of a subclinical outcome and did not alter the primary outcome (*n* = 5). It nonetheless illustrates that a normalized follow-up calprotectin does not remove the need for ongoing clinical vigilance for new-onset digestive symptoms, and constrains any claim that the negative predictive value of the overall screening strategy—as opposed to the specific, endoscopically verified subgroup D pathway—is 100%.

Capsule endoscopy was performed after dietary preparation alone, in accordance with the standard protocol of our unit, whereas current ESGE guidance recommends purgative-based regimens to optimize mucosal visualization. This may have reduced small-bowel visualization quality and could theoretically have led to underdetection of subtle mucosal lesions, although it reflects real-world practice in our setting.

Finally, the observational design precludes definitive conclusions about the causal directionality of the association between immunosuppression and subclinical ileitis discussed above, and does not entirely rule out the existence of an unmeasured confounder explaining both phenomena simultaneously.

## Conclusion

In conclusion, this exploratory prospective cohort provides proof-of-concept evidence that a persistently elevated fecal calprotectin trajectory may identify a subgroup of patients with idiopathic uveitis or scleritis harboring subclinical ileitis, a clinically relevant finding in a population without digestive symptoms at inclusion. From an ophthalmological perspective, these results suggest that, in patients with idiopathic ocular inflammation and persistently elevated fecal calprotectin, the absence of gastrointestinal symptoms does not rule out subclinical intestinal involvement. However, because endoscopic verification was performed only in patients selected by the fecal calprotectin-based protocol, this study does not establish that fecal calprotectin can reliably exclude subclinical intestinal disease, nor does it validate a screening algorithm for routine etiological work-up. This hypothesis is supported by a consistent pathophysiological framework centered on the gut-eye axis and by convergent external evidence regarding the directionality and genetic substrate of this association; nevertheless, validation in larger, prospectively and systematically verified multicenter cohorts is required before routine or targeted screening can be recommended.

## Data Availability

The raw data supporting the conclusions of this article will be made available by the authors, without undue reservation.
