# Supplementary material for: Serial fecal calprotectin as a screening tool for subclinical ileitis in patients with idiopathic uveitis or scleritis: a prospective cohort study
Source: Front Med (Lausanne). 2026 Oct 26;13:1968285. doi: 10.3389/fmed.2026.1968285 (PMC13745227; doi:10.3389/fmed.2026.1968285)
Supplement: Supplementary file 1 [file Datasheet1.pdf]

SUPPLEMENTARY TABLE 1 | Additional baseline characteristics of the study population (N=69).

Variable	Value	Valid n
**General demographic and clinical data**		
Charlson comorbidity index	0 (0–1)	69
Other immune-mediated disease, yes	9 (13.0%)	69
**Laboratory parameters**		
Hemoglobin, g/dL	14.3 ± 1.4	68
Leukocytes, $\times 10^3$/μL	6.30 (5.40–7.43)	68
ESR, mm/h	11 (6–20)	65
Rheumatoid factor, positive/negative	2 (3.1%) / 62 (96.9%)	64
**Ocular characteristics**		
Anterior segment complications, yes	26 (37.7%)	69
Posterior segment complications, yes	29 (42.0%)	69

ESR, erythrocyte sedimentation rate. Quantitative variables are expressed as mean ± standard deviation or median (interquartile range), depending on their distribution (Shapiro-Wilk or Kolmogorov-Smirnov test, as appropriate); qualitative variables are expressed as absolute frequency and percentage.

SUPPLEMENTARY TABLE 2 | Additional comparison of baseline characteristics by baseline fecal calprotectin group (<50 vs ≥50 μg/g).

Variable	<50 μg/g (n=43)	≥50 μg/g (n=26)	p-value
Charlson comorbidity index	0.00 (0.00–1.00)	0.00 (0.00–1.00)	0.988
Other immune-mediated disease, yes	4 (9.3%)	5 (19.2%)	0.282
NSAID use, yes	4 (9.3%)	5 (19.2%)	0.282
PPI use, yes	19 (44.2%)	10 (38.5%)	0.641
Hemoglobin, g/dL	14.5 ± 1.2	14 ± 1.6	0.189
Leukocytes, $\times 10^3$/μL	6.20 (5.33–7.38)	6.65 (5.58–8.15)	0.357
ESR, mm/h	11 (4–18)	11 (7–21)	0.584
Rheumatoid factor, positive	2 (4.7%)	0 (0.0%)	0.523
IOP, mmHg	14.81 ± 2.98	13.81 ± 3.41	0.219

NSAID, non-steroidal anti-inflammatory drug; PPI, proton pump inhibitor; ESR, erythrocyte sedimentation rate; IOP, intraocular pressure. Quantitative variables were compared using Welch's t-test or the Mann-Whitney U test, depending on their distribution (Shapiro-Wilk test), qualitative variables, using Pearson's χ^2 test or Fisher's exact test, as appropriate. Group sizes reflect the corrected baseline fecal calprotectin allocation. No comparison reached statistical significance.

SUPPLEMENTARY TABLE 3 | Individual SUN uveitis course, HLA-B27, and NSAID/PPI status in the 57 evaluable patients without confirmed subclinical ileitis.

Case	SUN uveitis course	HLA-B27	NSAID use	PPI use	Baseline FC (µg/g)	Second FC (µg/g)	Clinical-protocol subgroup
6	Chronic	Negative	No	No	10.6	6.6	A
7	Recurrent	Negative	No	Yes	7.5	41.5	A
8	Recurrent	Not performed	No	No	5.0	12.7	A
9	Recurrent	Negative	Yes	No	16.5	16.5	A
10	Chronic	Negative	No	No	27.9	18.6	A
11	Recurrent	Not performed	No	No	48.5	17.0	A
12	Recurrent	Not performed	No	No	6.4	6.5	A
13	Recurrent	Negative	No	No	8.7	5.5	A
14	Recurrent	Positive	No	No	38.6	18.4	A
15	Persistent	Negative	Yes	Yes	5.0	5.0	A
16	Recurrent	Negative	Yes	Yes	6.4	5.0	A
17	Chronic	Negative	No	No	5.3	5.8	A
18	Recurrent	Negative	No	Yes	31.7	24.2	A
19	Chronic	Negative	No	Yes	17.5	37.5	A
20	Recurrent	Negative	No	No	14.3	13.4	A
21	Recurrent	Negative	No	No	5.2	5.0	A
22	Recurrent	Negative	No	Yes	38.7	21.0	A
23	Recurrent	Not performed	No	No	6.2	19.9	A
24	Persistent	Positive	No	Yes	5.0	14.6	A
25	Persistent	Negative	No	No	10.0	10.0	A
26	Recurrent	Not performed	No	No	11.5	11.5	A
27	Recurrent	Negative	No	No	5.5	5.0	A
28	Recurrent	Negative	No	Yes	49.7	5.0	A
29	Recurrent	Negative	No	Yes	7.8	13.1	A
30	Recurrent	Negative	No	No	37.0	49.0	A
31	Persistent	Negative	No	Yes	46.5	46.5	B
32	Persistent	Negative	No	Yes	10.2	100.0	B
33	Persistent	Not performed	No	Yes	38.8	58.9	B
34	Recurrent	Not performed	Yes	Yes	22.5	189.0	B
35	Persistent	Negative	No	Yes	29.8	52.8	B
36	Recurrent	Negative	No	No	30.5	53.0	B
37	Recurrent	Negative	No	No	18.0	156.0	B
38	Persistent	Negative	No	No	42.6	106.0	B
39	Recurrent	Negative	No	Yes	8.0	80.2	B
40	Persistent	Negative	No	Yes	26.4	26.4	B
41	Recurrent	Negative	No	Yes	8.7	54.0	B
42	Persistent	Not performed	No	No	54.1	12.1	C
43	Chronic	Negative	No	No	72.0	11.5	C
44	Persistent	Not performed	No	No	168.0	5.9	C
45	Persistent	Negative	No	Yes	66.0	46.4	C
46	Persistent	Not performed	No	No	158.0	43.8	C
47	Recurrent	Not performed	Yes	Yes	97.0	16.3	C
48	Persistent	Negative	No	No	74.8	24.7	C
49	Persistent	Negative	No	No	81.7	5.2	C
50	Recurrent	Negative	No	No	116.0	36.8	C
51	Recurrent	Negative	No	Yes	183.0	29.4	C
52	Persistent	Negative	No	No	84.1	5.9	C
53	Chronic	Not performed	No	No	65.7	54.0	D
54	Recurrent	Negative	Yes	No	88.8	88.8	D
55	Recurrent	Negative	No	Yes	202.0	96.5	D
56	Recurrent	Negative	No	No	177.0	94.8	D
57	Chronic	Negative	Yes	Yes	92.7	98.5	D
58	Persistent	Negative	No	Yes	660.0	N/A	D
59	Recurrent	Negative	Yes	Yes	72.0	212.0	D
60	Recurrent	Not performed	Yes	No	136.0	94.5	D
61	Recurrent	Negative	No	Yes	109.0	204.0	D
62	Recurrent	Negative	No	Yes	61.8	466.0	D

FC, fecal calprotectin; HLA-B27, human leukocyte antigen B27; NSAID, non-steroidal anti-inflammatory drug; PPI, proton-pump inhibitor; SUN, Standardization of Uveitis Nomenclature; N/A, not available. Clinical-protocol subgroups: A, both FC determinations <50 µg/g; B, first <50 µg/g and second ≥50 µg/g; C, first ≥50 µg/g and second <50 µg/g; D, persistent FC elevation or baseline FC ≥250 µg/g. Cases 1-5 with confirmed subclinical ileitis are reported in Table 6; one patient with pending digestive evaluation is not included. Case numbering is for reference only and does not correspond to Record ID.

SUPPLEMENTARY TABLE 4 | Additional comparison of baseline characteristics by presence or absence of confirmed subclinical ileitis.

Variable	Ileitis (n=5)	No ileitis (n=57)	p-value
Charlson comorbidity index	0 (0–0)	0 (0–1)	0.454
Other immune-mediated disease, yes	1 (20.0%)	8 (14.0%)	0.557
NSAID use, yes	0 (0.0%)	9 (15.8%)	1.000
PPI use, yes	1 (20.0%)	26 (45.6%)	0.376
Hemoglobin, g/dL	14.10 (13.80–14.30)	14.00 (13.20–15.00)	0.979
Leukocytes, ×10³/µL	6.60 (6.20–7.10)	6.30 (5.30–7.40)	0.689
ESR, mm/h	11 (11–21)	11 (6–20)	0.733
Rheumatoid factor, positive	0 (0.0%)	1 (1.8%)	1.000
Anterior segment complications, yes	2 (40.0%)	22 (38.6%)	1.000
Posterior segment complications, yes	1 (20.0%)	25 (43.9%)	0.388

NSAID, non-steroidal anti-inflammatory drug; PPI, proton pump inhibitor; ESR, erythrocyte sedimentation rate. Quantitative variables are compared using Welch's t-test or the Mann-Whitney U test, depending on their distribution; qualitative variables, using Fisher's exact test. Seven patients were excluded from this outcome-based analysis: six who were not classifiable into the clinical-protocol subgroups and one with pending endoscopic evaluation. No comparison reached statistical significance.
